# Age and Gender-Specific Reference Intervals for Uric Acid Level in Children Aged 5–14 Years in Southeast Zhejiang Province of China: Hyperuricemia in Children May Need Redefinition

**DOI:** 10.3389/fped.2021.560720

**Published:** 2021-11-10

**Authors:** Chengjun Dai, Chaoban Wang, Fangqin Xia, Zishuo Liu, Yiqi Mo, Xiaoou Shan, Yonghai Zhou

**Affiliations:** The Second Affiliated Hospital and Yuying Children's Hospital of Wenzhou Medical University, Wenzhou, China

**Keywords:** uric acid, children, reference interval, Southeast China, body mass index

## Abstract

**Context:** Hyperuricemia is defined when the plasma uric acid concentration is above 416 μmol/L (7 mg/dl) in male adults, or 357 μmol/L (6 mg/dl) in female adults. However, there are no explicit criteria yet for children.

**Objective:** It is necessary to set up reference intervals for the uric acid level in different age groups among children.

**Materials and Methods:** A total of 5,439 individuals (3,258 males, 2,181 females) were included in the final statistical analysis. Reference values of all age groups were determined by statistical descriptions. Multiple linear regression analysis was applied to determine the relationship between uric acid level, BMI, and age.

**Results:** The level of uric acid increased with age. Gender differences in uric acid level occurred after the onset of puberty. Additionally, linear regression revealed a positive correlation between the uric acid level and BMI.

**Discussion and Conclusion:** The reference range of the uric acid level in children is inconsistent with the previous viewpoint. Body mass index plays an important role in uric acid metabolism.

## Introduction

Uric acid is the end product of purine nucleotides metabolism, which has the function of scavenging oxygen radicals, protecting the cell membrane, and stabilizing vascular endothelium. A high level of uric acid in the blood is called hyperuricemia. Hyperuricemia has been observed to cause insulin resistance and further metabolic syndromes (1). Therefore, blood uric acid level is closely related to hypertension, diabetes, and dyslipidemia (2, 3). For children, it is necessary to keep the uric acid level in a normal range.

However, the definition of hyperuricemia, plasma uric acid concentration >416 μmol/L (7.0 mg/dl) in male adults and >357 μmol/L (6.0 mg/dl) in female adults (4), remains unchanged for decades. There are no appropriate criteria to define the normal uric acid level in healthy children. It has been proved that the general serum uric acid (SUA) level of children rises gradually from infancy to adolescence, and the fastest increase of SUA occurs in puberty (5). Hormonal changes are cited as the reason for this trend. Therefore, it is of great significance to establish age-and-gender-specific reference intervals for the uric acid level in children.

Additionally, the relationship between hyperuricemia and obesity has been elucidated (6). It can be inferred that in healthy children, the serum uric acid level may increase with body mass index (BMI). Nevertheless, studies on the quantitative relationship between BMI and uric acid are rare. Further research in this field will be helpful for the growth of children.

## Materials and Methods

### Study Population

This study was approved by the ethics committee of the Second Affiliated Hospital and Yuying Children's Hospital of Wenzhou Medical University (Ethics approval number LCKY2019-289). The subjects were selected from the children who had physical check-ups in the hospital. A total of 13,708 participants aged 5–14 years (7,658 males and 6,360 females) who met the inclusion criteria were included and the informed consents were signed by their parents/legal guardians. These participants received physical examinations (including height, body weight) and blood tests (including routine blood tests, blood biochemistry tests, and lipid profile tests) during 2013–2018. They did not have any diagnosis of acute diseases within 2 weeks or chronic diseases. None of them had a medication intake within 2 weeks before taking medical examinations. To ensure that every child met the standard of health as much as possible, exclusion criteria were established and listed below:

1. Any data discrepancy.
2. White blood cell <4^*^10^∧^9/L or >12^*^10^∧^9/L.
3. Dyslipidemia (triglyceride >1.76 mmol/L, low density lipoprotein >3.38 mmol/L, cholesterol >5.2 mmol/L, high density lipoprotein <1.04 mmol/L).
4. Alanine aminotransferase (ALT) >40 U/L.
5. BMI <5th percentile or >95th percentile of the same age and same gender.

According to the exclusion criteria, there were 5,506 apparently healthy individuals (3,393 males and 2,270 females) remain in our study.

### Sample Processing and Testing

All participants were asked to fast overnight (at least 8 h) and sit for at least 30 min before blood samples collection. Blood samples were collected from a brachial vein and centrifuged within half an hour. All blood samples were tested in the clinical laboratory at the Second Affiliated Hospital and Yuying Children's Hospital of Wenzhou Medical University. SUA, ALT, and lipid profile were analyzed by Siemens ADVIA 2400 automatic biochemical analyzer (Siemens, Germany), routine blood tests were analyzed by Sysmex XE5000 automatic hematological analyzer (Sysmex, Japan). Tanner stage was adopted as the diagnostic criteria for puberty, which is breast bud palpable under the areola for girls, and the volume of the testis is more than 4 ml for boys.

### Statistical Analysis

All statistical analyses were performed by PASW Statistics 18.0 (SPSS Inc, Chicago, IL, USA). The distribution of the data was detected using the Kolmogorov-Smirnov test. Histograms and scatter plots were used to check the distributions. Data of normal distribution were expressed by means and standard deviation. The 95th percentile of SUA for each group was determined separately for males and females as age and gender-specific reference intervals. Independent–samples *T*-test was used to determine the difference between the genders. Multivariate analysis of variance was used to analyze the difference of uric acid in children of different ages and genders. Multiple linear regression was used to determine the relationship between uric acid, BMI and age, and outliers were identified by calculating the residuals. We analyzed the cutoff points of uric acid before and after puberty using the ROC curve. The outlier was removed if the residual was >3 times above or below the maximum and minimum, respectively.

## Results

**Figure 1:**
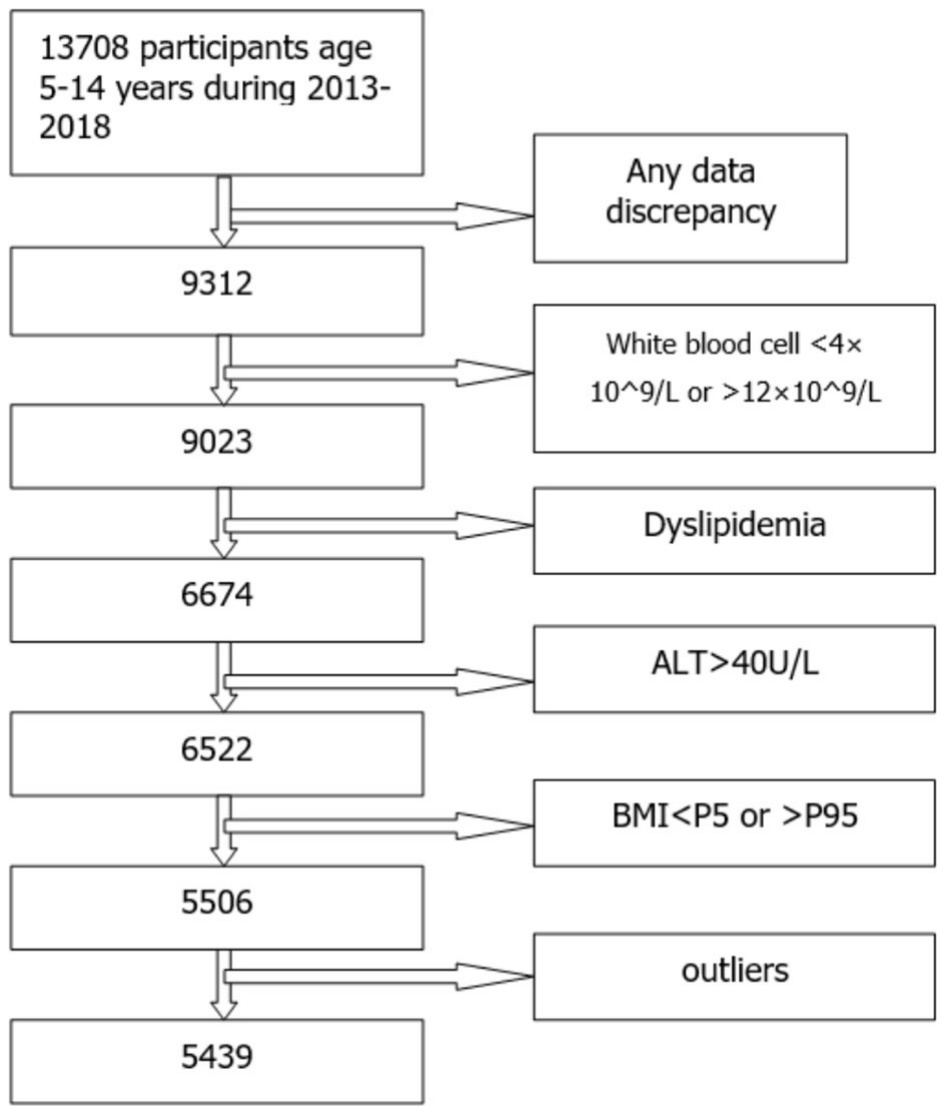
The selection of participants.

**Figure 2:**
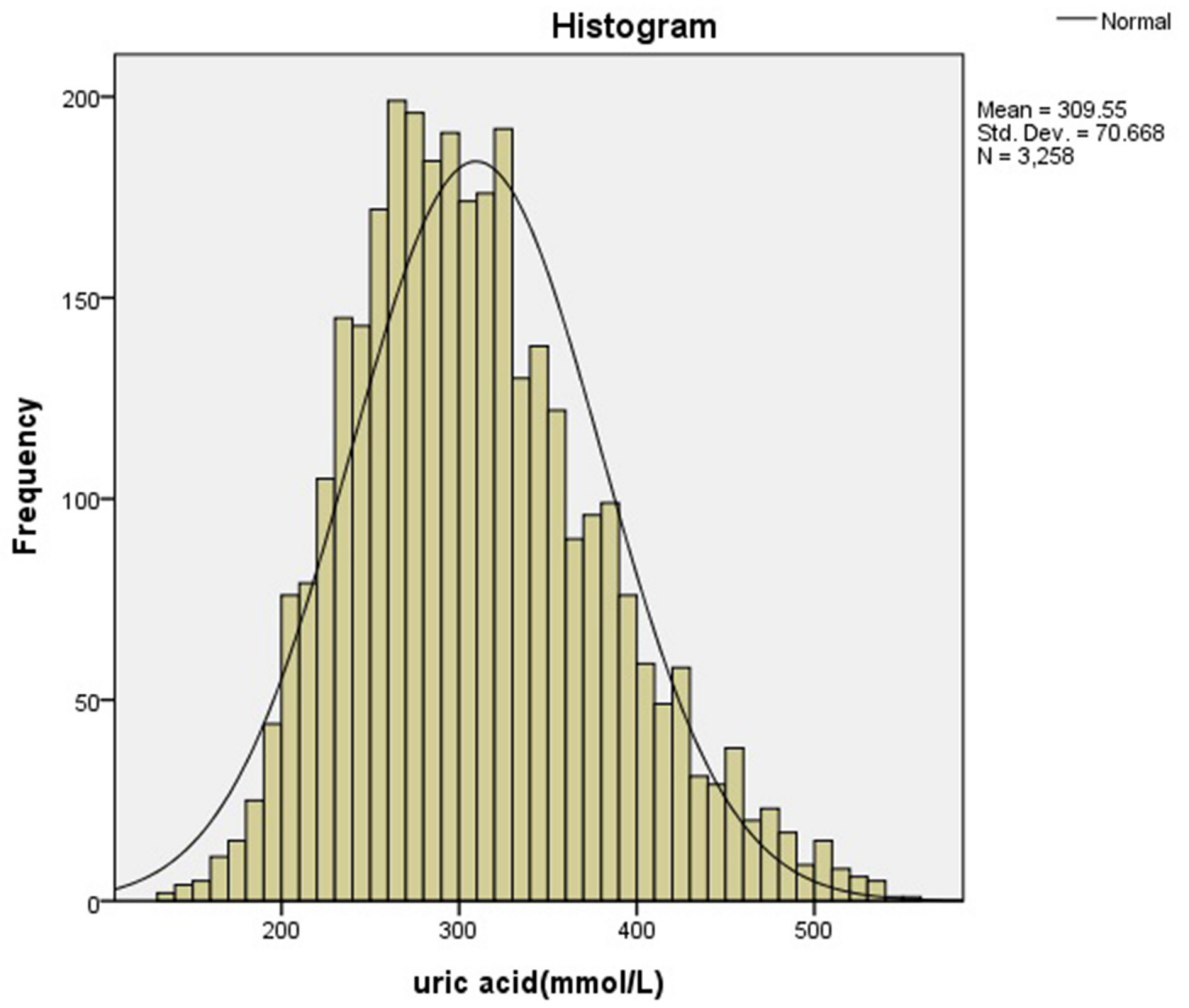
The distribution of uric acid in males.

**Figure 3:**
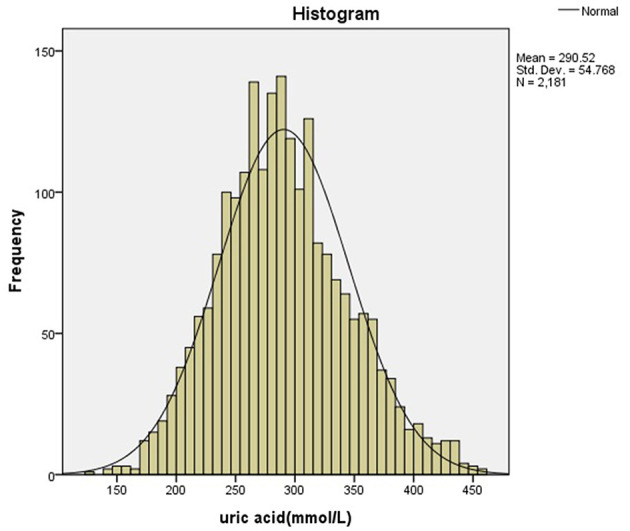
The distribution of uric acid in females.

After removing the outliers, a total of 5,439 individuals (3,258 males, 2,181 females) were included in the final statistical analysis. Figure 1 showed the selection of participants. Figures 2, 3 showed the distribution of uric acid in males and females. We divided the participants into 20 groups according to different ages and genders. Statistical results showed that the uric acid of males and females were all in the normal distribution. The sample size, means, standard deviation, and 95% reference intervals for each group were shown in Table 1. Statistical results showed that there was no difference in SUA between boys and girls before the age of 10, but the difference occurs after 10 years old.

**Table 1:** Mean, Std., and 95% reference intervals for uric acid (μmol/L).

**Age (Y)**	**Females**					**Males**					
	***N***	**Mean**	**Std**.	**P2.5**	**P97.5**	***N***	**Mean**	**Std**.	**P2.5**	**P97.5**	***P* ^a^**
5	166	276.83	53.276	172.4	381.3	285	283.38	57.902	169.9	396.9	*P* > 0.05
6	245	278.22	50.01	180.2	376.2	346	280.32	52.89	176.7	384	*P* > 0.05
7	256	279.76	50.667	180.5	379.1	332	282.82	57.495	170.1	395.5	*P* > 0.05
8	347	283.31	51.964	181.5	385.2	393	289.45	58.232	175.3	403.6	*P* > 0.05
9	330	293.12	56.155	183.1	403.2	409	290.5	58.757	175.3	405.7	*P* > 0.05
10	280	289.31	53.15	185.1	393.5	388	304.34	61.885	183	425.6	^*^P < 0.05
11	211	299.61	56.801	188.3	410.9	399	323.24	68.794	188.4	458.1	^*^P < 0.05
12	161	310.52	54.461	203.8	417.3	322	343.77	72.543	200.9	487.8	^*^P < 0.05
13	118	314.25	52.737	210.9	417.6	235	379.78	71.06	240.5	519.1	^*^P < 0.05
14	67	321.73	59.843	204.4	439	149	384.63	72.742	240.4	530.1	^*^P < 0.05

a *The p-values were derived from the independent–samples T-test*.

* *Means statistically significant*.

**Figure 4:**
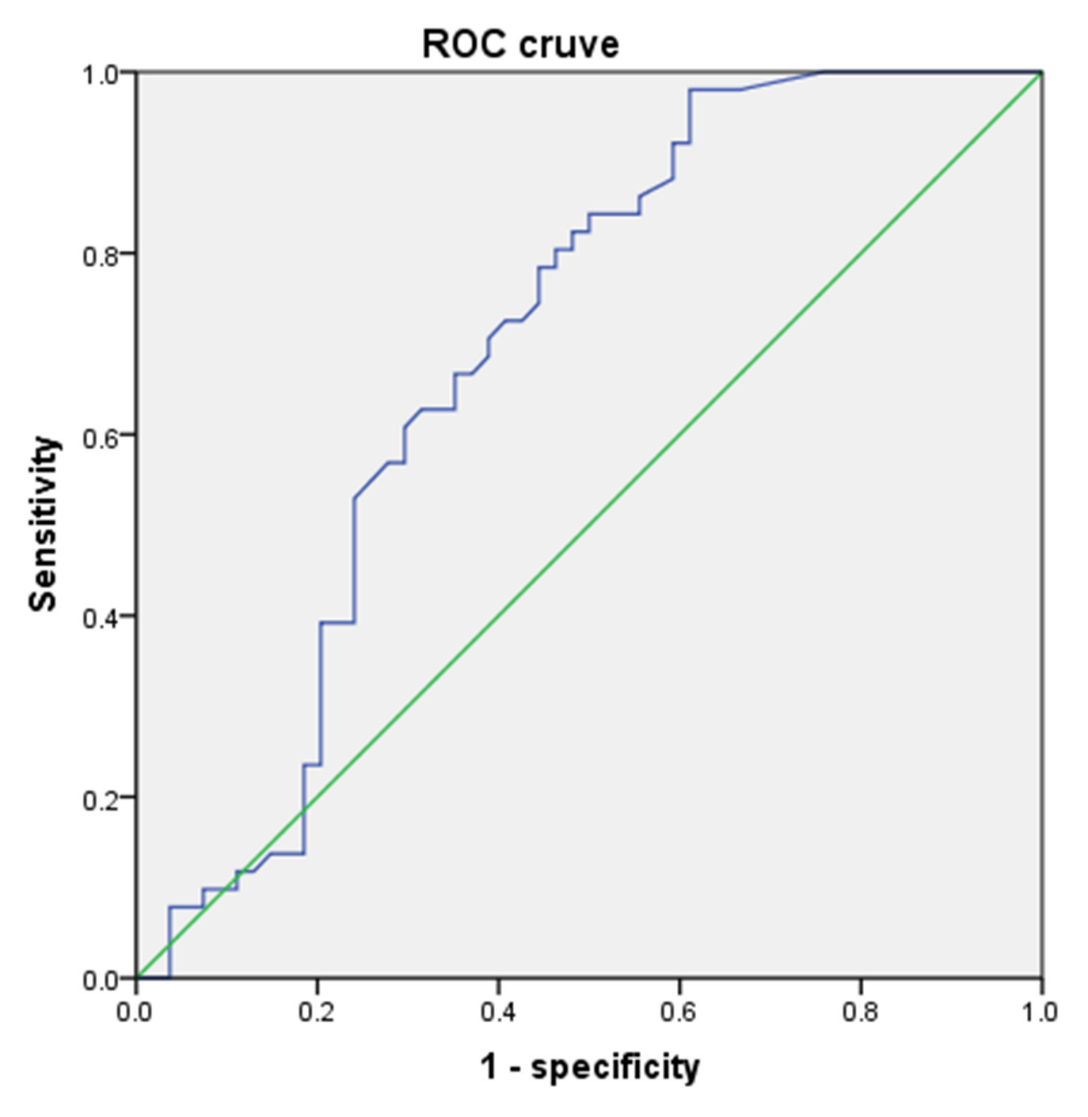
The ROC curve of uric acid to predict puberty in males.

**Figure 5:**
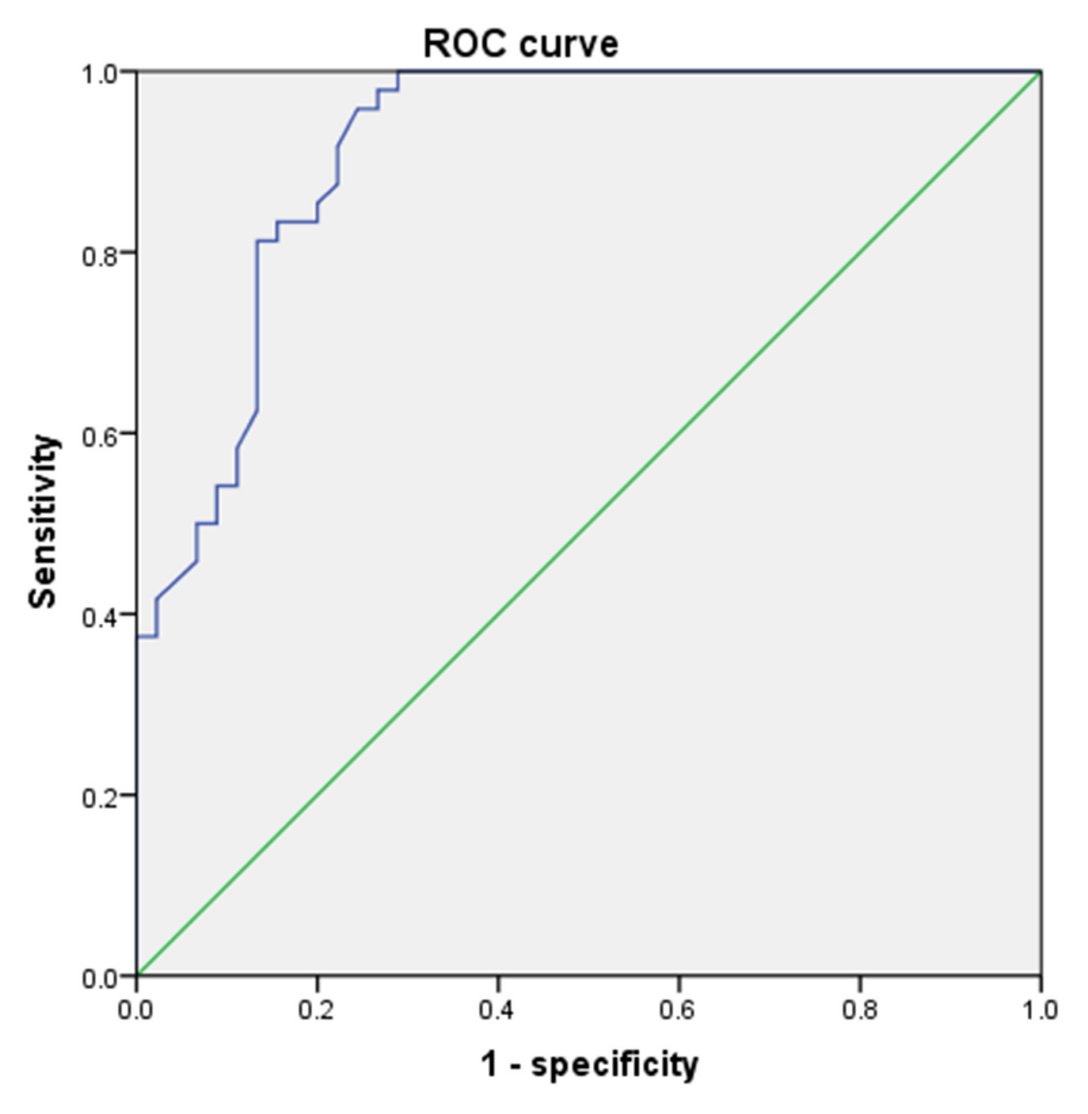
The ROC curve of uric acid to predict puberty in females.

According to multivariate analysis of variance, there were few differences in uric acid value among children of different ages and genders before the age of 9 years. The rapid increase of uric acid in males occurred at the age of 9–10, while it occurred at the age of 8–9 in females (according to independent–samples *T*-test, *P* < 0.05). According to the ROC curve, the girls' cutoff point is 230 μmol/L with a Youden index of 0.340, and 336 μmol/L for boys with a Youden index of 0.677. Figures 4, 5 showed the ROC curve.

**Figure 6:**
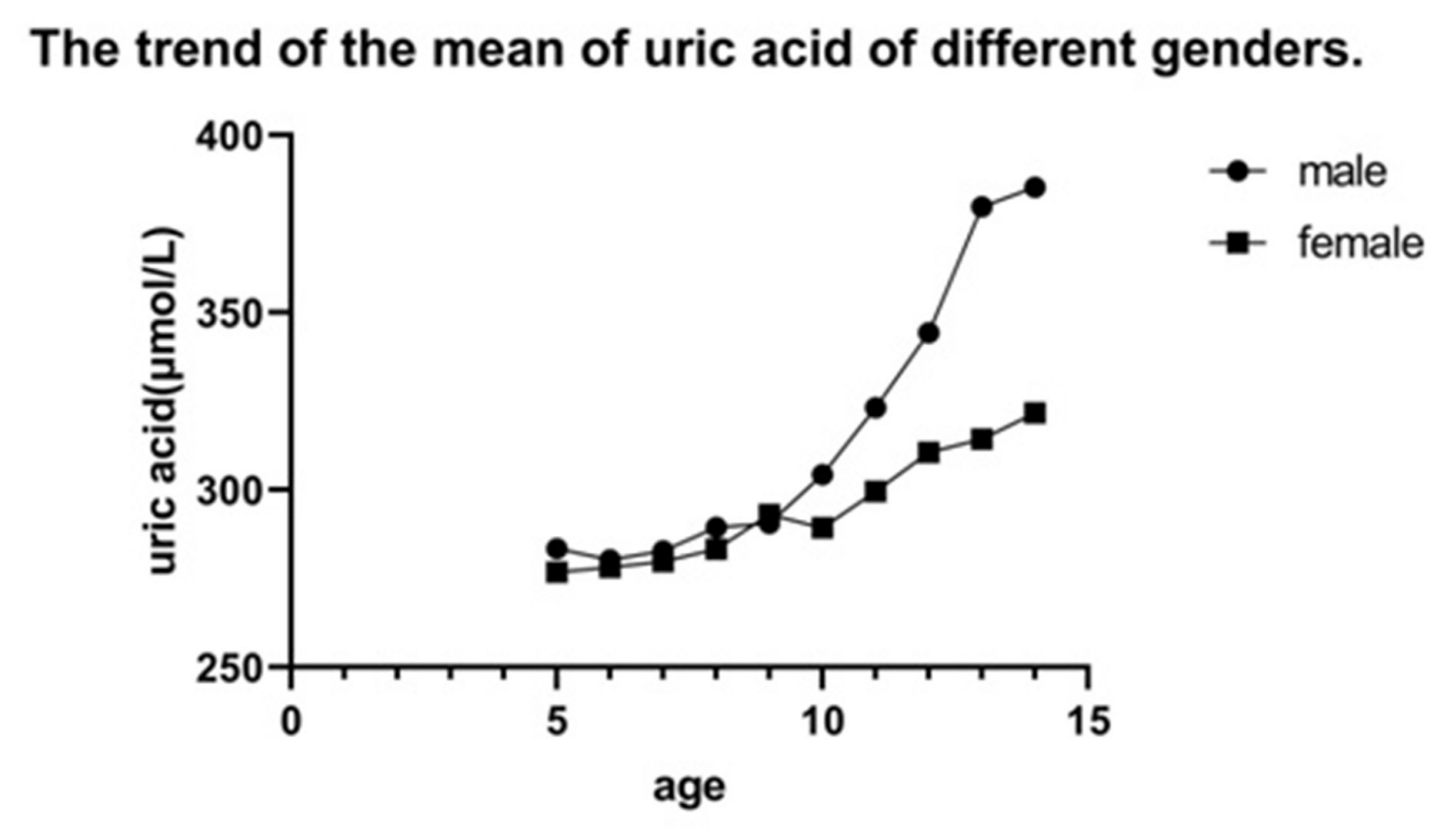
The trend of the mean of uric acid of different genders.

In China, precocious puberty can be diagnosed with secondary sexual characteristics in boys before 9 years old and girls before 8 years old (7). Among children between 8 and 10 years old, some children entered puberty, while others did not. Therefore, we randomly selected the subjects aged 8–10 and divided them into pre-puberty group and post-puberty group according to Tanner stage. Statistical analysis indicated that the uric acid level of children after puberty was significantly higher than that before puberty. Table 2 showed the change in the uric acid level before and after puberty. Before puberty, uric acid slowly increased with age. In contrast, blood uric acid levels increased rapidly during adolescence. Both boys and girls had this trend, but the increase of SUA was faster in boys. Figure 6 showed the trend of the mean of uric acid of different genders.

**Table 2:** The change of uric acid level before and after puberty (μmol/L).

	**Before puberty**	**After puberty**	***P***
Boys (*n* = 1,190)	284.53	329.02	<0.001
Girls (*n* = 957)	256.80	292.10	0.006

According to multiple linear regression analysis, we found that uric acid increased with age and BMI. Mean uric acid (μmol/L) in males was 109.321 + BMI ^*^ 7.69 +age (year)^*^ 7.76 (R^2^ = 0.216, *P* < 0.001), and 177.848 + BMI ^*^ 5.623 + age (year)^*^ 2.407 in females (R^2^ = 0.072, *P* < 0.001).

## Discussion

Uric acid is a very important index to evaluate renal function. Recent studies have confirmed that uric acid is closely related to obesity (8), non-alcoholic fatty liver disease (NAFLD) (2, 9, 10), hypertension (11, 12), and diabetes (1, 3). SUA can even affect the bone metabolism of teenagers (13). However, a uniform reference range for children's uric acid hasn't been established yet in the world. The difficulty is that uric acid is affected by lifestyle such as eating habits, so the reference intervals for each region will be different. Thus, it is of great significance for clinical work to establish age-and-gender-specific reference intervals of children's uric acid in different regions. And our results showed that children's uric acid seemed to be higher than the previous concept.

In our retrospective study, we found that uric acid increased year by year with the increase of age in 5–15 years old, which was a common feature of male and female groups. It might be due to the increase in muscle content and nutrition metabolism (14). However, the increase of uric acid was slow before 9 years old. Uric acid increased rapidly in girls at the age of 9 years and boys at the age of 10 years, and this was consistent with the development of puberty. In China, it's normal for girls to enter puberty after 9 years old, and 10 for boys (15).

Before puberty, SUA levels were almost the same between males and females. But after puberty, there was a difference in SUA between the genders, which was consistent with a study in the United States (16). The change of sex hormones during puberty might account for this phenomenon. During adolescence, androgens (especially testosterone) increase in the body. A study performed in Japan demonstrated that testosterone could promote the increase of uric acid (17). The mechanism is as follows: increased testosterone promotes muscle anabolism, while muscle mass is a major source of purine. Therefore, the increase of testosterone will lead to an increase in SUA. In addition, increased muscle mass will lead to increased adenosine triphosphate metabolism, and more purine intermediates in the muscle will be released, which can affect uric acid levels. On the other hand, testosterone can inhibit the excretion of uric acid while estrogen can promote the excretion of uric acid (18).

Our retrospective study found that the current reference intervals of uric acid in children in Southeast Zhejiang Province of China seemed to exceed our expectations. The 97.5th percentile of SUA of healthy boys after puberty was nearly 530 μmol/L, which was much more than 417 μmol/L for adult men. But the results obtained from relevant literature of the United States (16), Australia (19), and some other countries were similar to our research. We speculate that this might be related to the vigorous metabolism of puberty. In addition, as a coastal area, southeast Zhejiang Province of China has a higher seafood consumption than other areas, thus leading to a correspondingly higher SUA in the residents.

Through multiple linear regression, we found that uric acid increased with BMI. Although the coefficient was low, it can be proved that there is a positive correlation between uric acid and BMI. Insulin resistance might play an important role in this phenomenon (6).

There were still some limitations in our retrospective research. Some mixed factors, such as diet, exercise and other lifestyles were not completely unified. In addition, the level of serum uric acid may change with the season. All of these interfered with the results of this study. In future, we will conduct a cross-sectional study, and we will cooperate with more medical centers to obtain a more accurate reference interval. In addition, to distinguish between healthy and unhealthy, we established some inclusion and exclusion criteria, but the possibility that some “unhealthy” children were included in the study exists, for some patients may not have symptoms during the incubation period. For improvement, we will adopt stricter inclusion criteria in further research.

In conclusion, our study explored the growth trend of uric acid before and after puberty in detail. We also proved that there was a positive correlation between the uric acid level and BMI. This work was of great significance in guiding the healthy growth of children.

## Data Availability Statement

The raw data supporting the conclusions of this article will be made available by the authors, without undue reservation.

## Ethics Statement

The studies involving human participants were reviewed and approved by the Ethics Committee of the Second Affiliated Hospital and Yuying Children's Hospital of Wenzhou Medical University (Ethics approval number: LCKY2019-289). Written informed consent to participate in this study was provided by the participants' legal guardian/next of kin.

## Author Contributions

CD and CW performed the research and wrote the paper. XS and YZ designed the research study. FX and ZL analyzed the data. YM collected and screened the data. All authors contributed to the article and approved the submitted version.

## Conflict of Interest

The authors declare that the research was conducted in the absence of any commercial or financial relationships that could be construed as a potential conflict of interest.

## Publisher's Note

All claims expressed in this article are solely those of the authors and do not necessarily represent those of their affiliated organizations, or those of the publisher, the editors and the reviewers. Any product that may be evaluated in this article, or claim that may be made by its manufacturer, is not guaranteed or endorsed by the publisher.
